# One-year mortality of emergency department patients with substance-induced psychosis

**DOI:** 10.1371/journal.pone.0270307

**Published:** 2022-06-21

**Authors:** David Barbic, Madelyn Whyte, Gurwinder Sidhu, Allesandra Luongo, Tapash Apu Chakraborty, Frank Scheuermeyer, William G. Honer, Robert Stenstrom

**Affiliations:** 1 Department of Emergency Medicine, University of British Columbia, Vancouver, Canada; 2 Centre for Health Evaluation and Outcomes Sciences, Vancouver, Canada; 3 Faculty of Science, University of British Columbia, Vancouver, Canada; 4 Faculty of Medicine, University of British Columbia, Vancouver, Canada; 5 Department of Psychiatry, University of British Columbia, Vancouver, Canada; 6 BC Centre for Mental Health and Substance Use Service Research, Vancouver, Canada; Mayo Clinic Rochester: Mayo Clinic Minnesota, UNITED STATES

## Abstract

**Objectives:**

Psychosis is a well established complication of non-prescription drug use. We sought to measure the 1-year mortality of emergency department patients with substance-induced psychosis (SIP).

**Methods:**

This study was a multi-centre, retrospective electronic medical records review of patients presenting to the ED with substance-induced psychosis (SIP). We interrogated the hospital ED database from Jan 1, 2018 and Jan 1, 2019 to identify consecutive patients. All patients were followed for one year from index visit, and classified as alive/dead at that time. Patients were included in the study if they met the following criteria: 1) ED discharge diagnosis of psychosis NOS and a positive urine drugs of abuse screen (UDAS) or the patient verbally endorsed drug use, or 2) Mental disorder due to drug use and “disorganized thought”, “bizarre behavior” or “delusional behavior” documented in the chart and one or more of the following criteria: a) arrival with police, b) mental health certification, c) physical restraints, d) chemical restraints. We excluded patients who were not British Columbia residents, since we were unable to ascertain if they were alive or dead at 1 year from their index ED visit. Primary statistical analysis was logistic regression for risk of death in 1 year, based on plausible risk factors, selected a priori.

**Results:**

We identified 813 presentations for SIP (620 unique patients). The median age of the entire cohort was 35 years (IQR 28–44), and 69.5% (n = 565) were male. Thirty five patients (4.3%; 95% CI 3.2–5.9) had died one year after their initial presentation to the ED for SIP. Separate multivariable logistic regression analyses, controlling for age, demonstrated schizophrenia (OR 4.2, 95% CI 1.8–11.1) significantly associated with increased 1-year mortality.

**Conclusions:**

In our study of patients presenting to the ED with SIP, the 1-year mortality was 4.3%. Controlling for age, schizophrenia was a notable risk factor for increased 1-year mortality.

## Introduction

### Background

Non-prescription drug use is a significant public health issue in North America and globally [1, 2]. There were over 200,000 amphetamine related hospitalizations in the United States in 2015, with total health care costs of over $2 billion [3]. Between 2018 and 2021, cocaine and methamphetamines were the second and third most common drugs identified among persons with fatal drug overdose events in British Columbia [3], and recent data indicate that the burden of fatal drug overdoses has increased during the COVID-19 pandemic [4, 5].

### Importance

Psychosis is a long established complication of opioid, amphetamine and cannabis use [6, 7]. These patients are often initially assessed and treated in emergency departments (ED). Current evidence suggests that almost one fifth of all psychiatric presentations to an academic ED were related to amphetamine use [8, 9], yet the burden of substance-induced psychosis (SIP) on a national and global scale is unknown.

Almost 40% of patients with SIP will be discharged to the community from the ED after treatment with antipsychotics and rapid resolution of their symptoms [7]. While ED-based outcomes have been measured, there is little information on the subsequent mortality of these patients.

### Goals of this investigation

We sought to measure the 1-year mortality of ED patients with SIP. We also wished to identify risk factors associated with increased risk of mortality, and identify future areas of support and intervention for this vulnerable patient population.

## Methods

### Study design and setting

This study was a multi-centre, retrospective electronic medical records review of patients presenting to the ED with substance-induced psychosis.

XXXXXX Hospital is a tertiary care, urban, inner-city hospital with an annual census of 90,000 ED visits; many patients have unstable housing and acute and chronic mental health and substance use disorders. The site has psychiatric, internal medicine, and addictions consultation services. XXXXXX Hospital is an affiliated community center with 35 000 annual ED visits and a general internal medicine service. Both hospitals are part of the XXXXXX Health region, where six EDs receive 300 000 patients annually.

#### Data source

Both sites share an electronic medical record (Cerner Millenium, Cerner, Kansas City, MO) that includes computerized physician order entry, medication administration including dosages and timing, results of all investigations including lab, ECG and radiology, all consultation, admission, and progress notes, and ED and hospital discharge summaries and final diagnoses. The medical record is linked to the regional ED database and the XXXXXX Vital Statistics and these linkages have been previously validated [10].

### Selection of participants

We interrogated the hospital ED database from Jan 1, 2018 and Jan 1, 2019 to identify consecutive patients with the following ED ICD9 discharge diagnoses via the hospital ED database: psychosis (292.1, 292.2, 298.8 and the following non-coded descriptors: Mental disorder due to stimulants NOS; Mental disorder due to cocaine; Mental disorder due to cannabis; Mental disorder due to opioids; Mental disorder due to multiple drug use; Mental disorder due to Hallucinogens.

Patients were included in the study if they met the following criteria:

1. ED discharge diagnosis of psychosis NOS and a positive urine drugs of abuse screen (UDAS) or the patient verbally endorsed drug use, or
2. Mental disorder due to drug use and “disorganized thought”, “bizarre behavior” or “delusional behavior” documented in the chart and one or more of the following criteria: a) arrival with police, b) mental health certification, c) physical restraints, d) chemical restraints.

We excluded patients who were not British Columbia residents, since we were unable to ascertain if they were alive or dead at 1 year from their index ED visit.

#### Data

We adhered to previously established standards for retrospective electronic medical record reviews in emergency medicine [11]. Based off prior work, data were extracted for all ED investigations including vital signs, laboratory investigations, electrocardiograms, and diagnostic imaging [7, 9, 12, 13]. Data were extracted from clinical documentation from emergency physicians, consulting specialist physicians, nursing and other allied health professionals. Data were extracted using a standard, pilot-tested data collection form by three trained research assistants (RAs). All RAs received a minimum of 4 hours of in-person training, and attended monthly study meetings. All RAs were blinded to the study hypothesis and outcomes. Strict data collection standards were reviewed on a monthly basis, and missing data was managed with additional data collection efforts by the primary investigator and source documentation verification. A random sample of data (10%) was double collected for the following variables: age, gender, triage score, and ED diagnosis and checked by study investigators to ensure quality and completeness. All variables were inspected for outlier values, and these were re-evaluated by examining original data/charts.

#### Sample size calculation

The required sample size was based on the primary outcome of 1 year mortality. Based off recent work demonstrating a 5.4% one-year mortality for ED visits following opioid overdose, a 95% confidence interval around an estimated incidence of 4% for mortality within 1 year of index visit +/- 1.5% was used [14]. Based on this, 725 total patient visits were required [15].

### Outcomes

The primary outcome was 1-year mortality following an ED visit for SIP. The measure of interest is the cumulative incidence of death. We obtained this outcome by reviewing linked vital statistics data within the electronic medical record, for 1 year following the patient’s index visit.

#### Statistical analysis

The unit of analysis is the patient ED visit. Descriptive statistics including counts, means, medians and interquartile ranges were used to describe the data. For mortality only, the unit of analysis is the individual patient. Since the one-year mortality rate was predicted to be about 28–38 patients, risk factors were assessed with separate multivariable logistic regressions with only two predictors per model, in order to avoid model overfitting, and unstable odds ratio estimates. Odds ratio coefficients provided by logistic regression in these analyses are a reasonable approximation to the relative risk, because the outcome (death at 1 year) is not common.

Predictor variables were specified a priori solely on the basis of plausibility; No statistical model selection process was employed. Type of drug use reported was assessed to establish if any specific drug (opiate, cocaine, or other [methamphetamine, etc]) was associated with increased mortality. Also of interest was the risk associated with differing types of mental disorder (schizophrenia, bipolar, depression).

Due to the small number of outcomes and to avoid model overfitting, interactions are not assessed. Age was anticipated to be a potential confounder for mortality, so it as included in each separate model, along with one other predictor (please see Table 2). A Bonferroni correction was applied to each logistic regression, by calculating the 99% confidence interval (instead of the usual 95% CI), for each risk factor. We then developed a Kaplan Meier curve to explore the time between ED visit for SIP, and subsequent 1-year mortality [16].

### Ethical considerations

This study adheres to all tenants of the Declaration of Helsinki and the Tri-Council Policy Statement on the use of human subjects in research. This study was approved by the XXXXXXXX Research Ethics Board (H18-03349). The Research Ethics Board waived the requirement for informed consent for this study.

## Results

### Characteristics of study subjects

We identified 813 presentations for SIP between Jan 1, 2018 and Dec 31, 2019 (620 unique patients). The median age of the entire cohort was 35 years (IQR 28–44), and 69.8% (n = 568) were male. Thirty five patients (4.3%; 95% CI 3.2–5.9) had died one year after their initial presentation to the ED for SIP. The mean age was 33.8, 25 (71.4%) were male. Please refer to Table 1 for further demographic information.

**Table 1:** Index visit demographic data for patients alive versus dead at 1 year post index visit.

Variable	Alive at 1 year N = 778	Dead at 1 year (N = 35)
Mean age (SD)	36.4 (10.7) years	33.8 (11.1) years
Male Gender	543 (66.8%)	25 (71.4%)
CTAS* level 1 or 2	445 (57.2%)	21 (60%)
Brought by police (section 28)	224 (28.8%)	4 (11.4%
ED visit in < 30 days from index	339 (43.6%)	13 (37.1%)
Required physical restraints	209 (26.9%)	10 (28.5%)
Admitted to psychiatry service	368 (47.3%)	22 (62.9%)
Admitted to non-psychiatry service	14 (1.8%)	2 (5.7%)
Medical Comorbidities:		
HIV+	65 (8.35%)	2 (5.7%)
Hep C+	164 (21.1%)	8 (22.9%)
Diabetes Mellitus	33 (4.2%)	0 (0%)
Hypertension	29 (3.7%)	0 (0%)
Coronary artery disease	4 (0.5%)	1 (3.9%)
COPD	25 (3.2%)	2 (5.8%)
Psychiatric Comorbidities:		
Schizophrenia	171 (22%)	19 (54.3%)
Bipolar disorder	109 (14%)	6 (17.1%)
Depression	85 (10.9%)	9 (25.7%)
Homeless	377 (48.5%)	11 (31.4%)
Endorsed using amphetamine	474 (60.9%)	19 (54.3%)
Endorsed using opiates	226 (29%)	15 (42.9%)
Endorsed using cocaine	95 (12.2%)	8 (22.9%)
Had labs ordered	646 (83%)	32 (91.4%)
Had Imaging ordered	120 (15.4%)	5 (14.3%)
Had ECG ordered	348 (44.7%)	20 (57.1%)

* CTAS = Canadian Triage and Acuity Scale.

Mean (SD) or N (%).

The inter-rater reliability of data extraction for age, gender, triage score, and ED diagnosis was κ = 0.83 (95% CI 0.78–0.88).

### Main results

**Fig 1:**
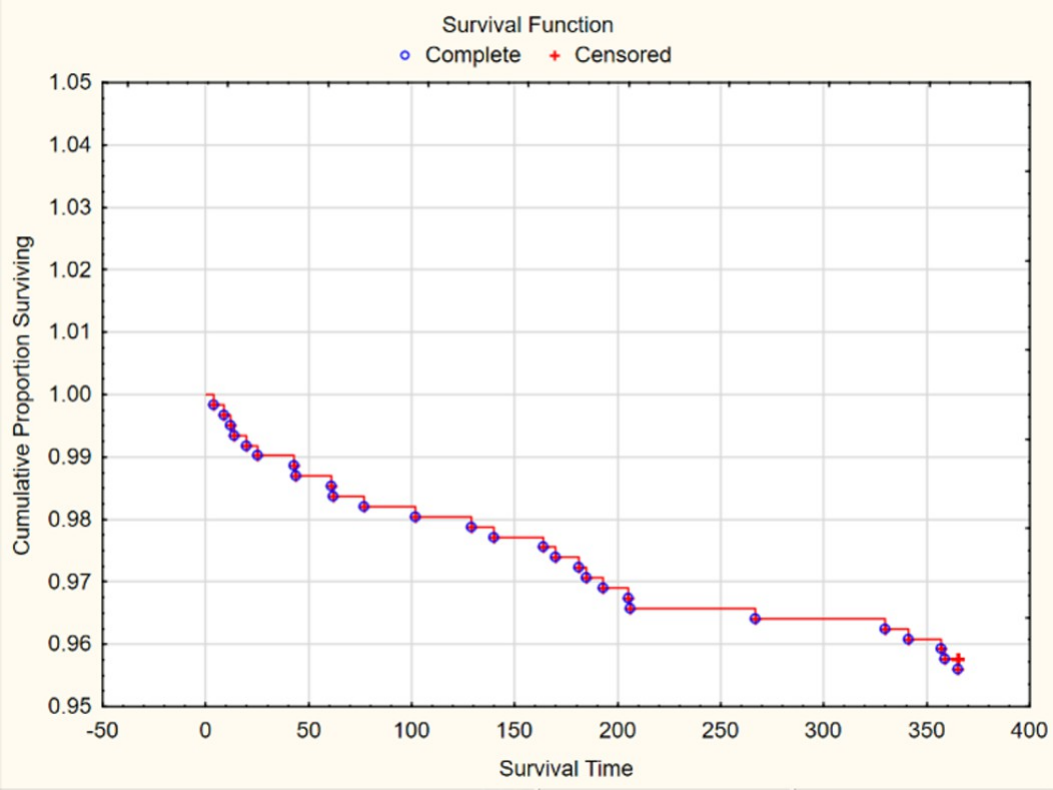
Kaplan Meier curve showing the proportion of study participants surviving from index visit (day 0) to 1 year (day 365).

Seven patients died within 30 days of the index SIP visit. Of those patients not alive at 1 year, 80% (n = 28) died between 30 days and 365 days after their initial presentation to the ED [Fig 1].

We conducted separate multivariable logistic regression analyses (univariable for the first predictor–age), controlling for age, to explore variables, based on biologic plausibility, associated with 1 year mortality in this cohort [Table 2]. The only significant predictor of 1 year mortality was a prior diagnosis of schizophrenia.

**Table 2:** Multivariable logistic regression analysis results– 1 year mortality.

Variable	Referent	OR	99% CI*
Age	--	1.02/year ↑	0.75–1.38/yr
Gender	Female	1.19	0.41–3.42
Opiate use	Negative	1.61	0.33–7.47
Cocaine/crack use	Negative	1.92	0.83–4.71
Amphetamine use	Negative	0.98	0.31–3.88
Homeless	Not homeless	0.48	0.17–1.33
Schizophrenia	No schizophrenia	4.2	1.82–11.05
Mood disorder	No mood disorder	1.27	0.38–4.12

• Each risk factor above represents a separate logistic regression [LR] with age (2 predictors per LR). In order to account for multiple models, we calculated the 99% confidence interval.

## Limitations

Our study is not without limitations. It is feasible that we may have missed some patients with substance induced psychosis (SIP) presenting to our EDs with the diagnostic codes used to identify cases in our study. In addition, it is conceivable that some of the patients included in our study who were admitted to hospital under the psychiatry service, were experiencing exacerbations of pre-existing mental health conditions such as schizophrenia or bipolar disorder, confounded by substance use. We attempted to control for this by reviewing the discharge documentation for the most responsible psychiatric diagnosis from the treating psychiatrist. It is possible that we may have lost some patients to follow up and were unaware of their true mortality at one year post index visit for SIP. Patients may have left the province of XXXXXXX and as a result, any deaths would not be reported to the XXXXXX Vital Statistics registry. However, we successfully obtained evidence of patients being alive at 1-year via documented encounters with the health care system for 93% of patients.

## Discussion

The burden of substance-induced psychosis (SIP) on a national and global scale is currently unknown. In this retrospective cohort of 620 unique patients presenting to two emergency departments (EDs) with SIP, we observed a 1-year crude mortality risk of 4.3% (95% CI 3.2–5.9).

Prior work in this area has failed to explore the 1-year mortality of these vulnerable patients. Using data linkage, Fatovich *et al*. reported a 2.9% risk of mortality associated with any visit to an ED related to amphetamine use [13], but this study did not specifically examine visits for acute psychosis. Additional work with a closely related patient population has demonstrated a 5.4% one year mortality associated with an ED visit for opioid overdose [14]. In our study, when controlling for age, patients endorsing opioid use (heroin or fentanyl), or with a positive urine drugs of abuse screen, did not demonstrate an increased risk of 1-year mortality [Table 2]. This finding stands in sharp contrast to the recent numbers of overdose deaths in our jurisdiction related to fentanyl contamination [17]. There are likely subtle differences between individuals in this population of persons that use drugs, which require additional research to better understand the risks contributing to premature mortality.

In our study, controlling for age, schizophrenia was a notable risk factor for increased 1-year mortality after an initial ED visit for SIP. It is unclear the exact mechanism of this increased risk, and it is likely related to multiple risk factors. Persons with schizophrenia are at increased risk of death by suicide [18], are more likely to be the victims of violent crimes [19], and experience increased mortality from cardiovascular disease compared to the general population [20]. These factors likely contribute to the increased 1-year mortality risk for persons with schizophrenia observed in our study, yet the magnitude of their contribution is unclear.

Homelessness is an independent risk factor for increased mortality [21, 22]. In our study, we did not observe a similar association. The reasons behind this are unclear, and may be related to our study being underpowered to clearly delineate this relationship. Our findings likely warrant further prospective study.

In conclusion, in our study of patients presenting to the ED with SIP, the 1-year mortality was 4.3%. Controlling for age, schizophrenia was a notable risk factor for increased 1-year mortality. These findings highlight an important area for improvements in care delivery and outreach opportunities to a highly marginalized and at risk population. Compassionate, trauma-informed care to address the co-existing mental health, addictions and social determinants of health challenges faced by these persons, may decrease their risk of 1-year mortality, and improve overall patient well-being.
